# Effects of miglustat treatment in a patient affected by an atypical form of Tangier disease

**DOI:** 10.1186/s13023-014-0143-3

**Published:** 2014-09-18

**Authors:** Annalisa Sechi, Andrea Dardis, Stefania Zampieri, Claudio Rabacchi, Paolo Zanoni, Sebastiano Calandra, Giovanna De Maglio, Stefano Pizzolitto, Valerio Maruotti, Antonio Di Muzio, Frances Platt, Bruno Bembi

**Affiliations:** Regional Coordinator Centre for Rare Diseases, University Hospital Santa Maria della Misericordia, Udine, Italy; Department of Biomedical; Metabolic and Neural Sciences, University of Modena and Reggio Emilia, Modena, Italy; Department of Pathology, University-Hospital Santa Maria della Misericordia, Udine, Italy; Centre for Neuromuscular Diseases, University G. d’Annunzio, Chieti, Italy; Department of Pharmacology, University of Oxford, Oxford, UK

**Keywords:** Tangier Disease, Niemann Pick type C, Miglustat, Glycosphingolipids

## Abstract

**Background:**

Tangier disease (TD) is a rare autosomal recessive disorder, resulting from mutations in the ATP binding cassette transporter (ABCA1) gene. The deficiency of ABCA1 protein impairs high density lipoprotein (HDL) synthesis and cholesterol esters trafficking.

**Case Report:**

A 58 year-old female, presenting with complex clinical signs (splenomegaly, dysarthria, dysphagia, ataxia, tongue enlargement, prurigo nodularis, legs lymphedema, pancytopenia and bone marrow foam cells), was misdiagnosed as Niemann-Pick C (NPC) and treated with miglustat (300 mg/day), normalizing neurological symptoms and improving skin lesions and legs lymphedema. Subsequently filipin-staining and molecular analysis for NPC genes were negative. Lipid profiling showed severe deficiency of HDL, 2 mg/dl (n.v. 45-65) and apoAI, 5.19 mg/dl (n.v. 110-170), suggesting TD as a probable diagnosis. Molecular analysis of *ABCA1* gene showed the presence of a novel homozygous deletion (c.4464-486_4698 + 382 Del). Miglustat treatment was then interrupted with worsening of some neurological signs (memory defects, slowing of thought processes) and skin lesions. Treatment was restarted after 7 months with neurological normalization and improvement of skin involvement.

**Conclusions:**

These results suggest miglustat as a possible therapeutic approach in this untreatable disease. The mechanisms by which miglustat ameliorates at least some clinical manifestations of TD needs to be further investigated.

**Electronic supplementary material:**

The online version of this article (doi:10.1186/s13023-014-0143-3) contains supplementary material, which is available to authorized users.

## Introduction

Tangier disease (TD) (OMIM #205400) is a rare autosomal recessive disorder, resulting from mutations in the ATP binding cassette transporter (*ABCA1*) gene, mapped to chromosome 9q22-q31. The *ABCA1* gene encodes a multiple trans-membrane domain protein (ABCA1) involved in the efflux of free cholesterol from peripheral cells to Apolipoprotein A-I (ApoAI) generating nascent high-density lipoprotein (HDL). As a result of the ABCA1 defect, patients present with a characteristic severe deficiency or absence of HDL in the plasma, rapid catabolism of ApoAI and an accumulation of cholesterol esters in macrophages and other reticuloendothelial cells in multiple tissues [1,2]. Biochemically, the disease is therefore characterized by very low plasma levels of HDL and ApoAI, low total cholesterol and normal or high levels of triglycerides. Classical clinical symptoms include hyperplastic orange tonsils, polyneuropathy, hepatosplenomegaly, enlarged lymph nodes, corneal clouding and premature atherosclerosis. Nevertheless, several atypical phenotypic presentations have also been described that include asymptomatic milder phenotypes, forms with early cardiac involvement and central nervous system presentation [3-5]. To date, there is no specific treatment for TD.

At the cellular level, several studies have suggested a homeostatic link between ABCA1 function and NPC1/NPC2, the two proteins involved in the transport of cholesterol and other lipids from late endosomes/lysosomes to other cellular compartments, including the endoplasmic reticulum [6,7]. The deficit of either NPC1 or NPC2 causes Niemann Pick C (NPC) disease (OMIM #257220, OMIM #607625), a rare autosomal recessive disorder characterized by the accumulation of unesterified cholesterol and other lipids within the late endocytic system of cells [8]. The expression of ABCA1 is impaired in human NPC1 disease fibroblasts. This translates into reduced HDL particle formation, providing a mechanism for the reduced plasma HDL cholesterol seen in the majority of NPC patients [6,9]. Clinically, NPC is characterized by progressive neurological deterioration and hepatosplenomegaly, with varying age at onset and ensuing course. While cholesterol is the main lipid accumulated in peripheral tissues in NPC disease, glycosphingolipids (GSL) are the major species that accumulate in the central nervous system [8]. Based on these findings, miglustat, an imino sugar drug that inhibits glucosylceramide synthase, the enzyme which catalyses the first step in GSL biosynthesis, has been evaluated for the treatment of neurological manifestations of NPC disease. Its efficacy was demonstrated first in animal models [10] and then in an international randomized clinical trial and in long-term extension studies [11-13]. Subsequently, miglustat was approved for the treatment of NPC in European Union countries, as well as most countries worldwide (e.g. Australia, Argentina, Canada, Russia).

In this manuscript we report the clinical features and the follow up of a patient with an atypical form of Tangier disease, who was initially misdiagnosed as NPC disease and treated with miglustat resulting in clinical benefit.

## Methods

### Filipin staining and NPC1-NPC2 molecular analysis

The filipin test for NPC disease was performed on cultured skin fibroblasts using the method described by Blanchette-Mackie et al. [14].

Molecular analysis of *NPC1, NPC2* was performed by PCR amplification of the exonic and intronic flanking regions, as previously described [15].

### Cell cholesterol efflux

Skin fibroblasts from patients with TD and healthy controls were used for the assay of ABCA1-mediated cholesterol efflux. Cell monolayers were incubated for 24 h in medium containing [1,2-^3^H]-cholesterol (2 μCi ml) and 1% fetal bovine serum. Following the 24 h labeling period, cells were washed and incubated overnight in media containing 0.2% bovine serum albumin, in the presence or absence of 9-cis-retinoic acid (5 μmol/l) and 22-hydroxycholesterol (10 μmol/l) to induce ABCA1 expression. [1,2-^3^H]-cholesterol-labeled monolayers were incubated for 6 h (efflux time) in the presence and in the absence of human Apo A-I (25 μg ml) as free cholesterol acceptor. Cholesterol efflux was quantified by measuring the radioactivity of the incubation medium after the removal of floating cells by centrifugation, using a time zero (To) set of cells to calculate total [^3^H]-cholesterol content in the monolayer. Fractional efflux was calculated as cpm [^3^H] in the medium ⁄ [^3^H] at T_0_ × 100. All the efflux assays were performed in triplicate [16].

### RNA extraction from fibroblasts and ABCA1 cDNA analysis

RNA was extracted using Eurozol (EuroClone; Celbio, Milan, Italy) and retrotranscribed to cDNA using SuperScript III (Invitrogen, Carlsbad, CA, USA), following the manufacturer’s instructions. To study the transcript of the mutant ABCA1 allele found in the proband (harboring a deletion of exons 32, 33 and 34) the cDNA was amplified using a forward primer (5′-TGCTGCCTGTGTGTCCCCCA-3′) complementary to exon 31 and a reverse primer (5-GGTTGGCCCGGAGAATGGCA -3′) complementary to exon 35, at the following amplification conditions: 95°C for 2 min and 95°C for 30 sec/ 63.4°C for 30 sec/ 72°C for 50 sec for 29 cycles followed by an extension at 72°C for 5 minutes. PCR products were separated on 4% agarose gel electrophoresis and sequenced [16].

### Ultrastructural analysis of the skin biopsy

A tissue sample of 1 mm^3^ was fixed in 2.5% glutaraldeide in 0.1 M cacodylate buffer for 4 hours at +4°C. Fixed tissue was then washed with cacodylate buffer 0.15 M and then postfixed with 1% osmium tetroxide in cacodylate buffer for 1 hour at +4°C. Tissue was dehydrated and embedded in Epon resin. Ultrathin sections of 70 nm were stained with uranyl acetate and Reynold′s lead citrate and examined with a Morgagni 286 transmission electron microscope (FEI Company).

### Case report

A 58 years old Caucasian female diagnosed with NPC disease was referred to the Regional Coordinator Centre for Rare Disease of Udine (RCCRD) in 2010. Her latest medical history (previous 2 years) had been characterized by progressive neurological symptoms: pain and hyposthenia of the left inferior leg followed by pain and hyposthenia of the left arm, psychomotor impairment, ataxic gait, tongue enlargement, dysarthria and dysphagia. Her medical history included mild thrombocytopenia and splenomegaly since the age of 32 years, dorsal skin itching nodular lesions resembling *prurigo nodularis* since the age of 40 years, and chronic lymphedema of the left leg since the age of 43 years. Electromyography (EMG) of the legs showed bilateral demyelinating motor neuropathy; brain magnetic resonance imaging (MRI) was negative. Haematological parameters showed the presence of pancytopenia (haemoglobin 10.2 g/dl, leukocytes 3700/ul, platelet 109000/ul), a bone marrow biopsy evidenced foam cells, interpreted as a lysosomal storage disorder. On the basis of clinical and histological data a diagnosis of NPC was made and therapy with miglustat (300 mg/day) was commenced.

After 4 months of therapy a regression of neurological symptoms, tongue enlargement, and left leg lymphedema was observed as well as a marked improvement of the skin lesions.

When the patient was for the first time admitted to RCCRD (after 6 months of miglustat therapy), her neurological examination was negative, skin lesions were very mild and both tonsils were normal in size and color. The EMG of upper and lower limbs was normal.

Specific diagnostic exams for NPC were then performed. Filipin test showed mild intracellular storage of unesterified cholesterol in cultured fibroblasts. However, no mutations were found in the *NPC1* or *NPC2* genes.

Lipid profiling showed the presence of severe hypocholesterolemia, total cholesterol 60 mg/dl (n.v. 130-200 mg/dl), very low levels of HDL-cholesterol, 2 mg/dl (n.v. 45-65 mg/dl) and apoAI, 5.19 mg/dl (n.v. 110-170 mg/dl) and hypertrigliceridemia, 448 mg/dl (n.v. 40-150 mg/dl). This profile was strongly suggestive for a diagnosis of TD, which was confirmed by molecular analysis.

**Figure 1:**
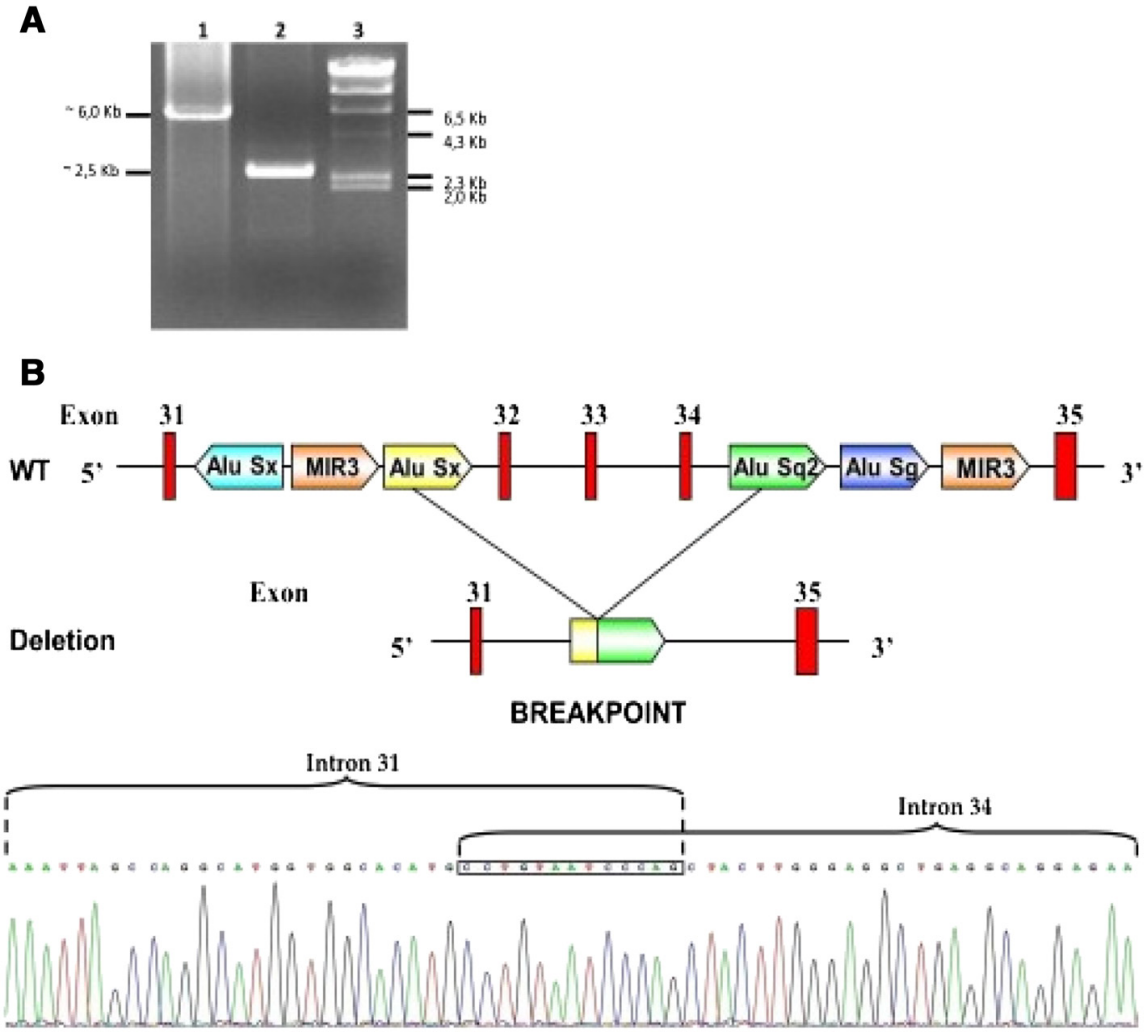
**Genetic analysis of the ABCA1 gene. A** PCR amplification of the *ABCA1* gene region spanning from exon 31 to exon 35 in patient # Mo.4 The figure shows the agarose gel electrophoresis of the PCR fragments obtained in a control subject (lane 1) and in the proband #Mo-4 (lane 2) respectively. Molecular size markers are shown in lane 3. The size of the band seen in lane 2 is consistent with a deletion of ~ 3.5 Kb. **B** Deletion of exon 32-34 of *ABCA1* gene found in patient #Mo-4. The upper panel shows Alu sequences located in intron 31 and intron 34. The *Alu* Sx in intron 31 and *Alu* Sq2 sequences in intron 34 are involved in the recombination event resulting in the deletion. The lower panel shows the nucleotide sequence of the genomic breakpoint indicating that the 3′ half of intron 31 is followed by the 5′ half of intron 34. The boxed nucleotide sequence is identical in the two introns and cannot be assigned to either of them.

**Figure 2:**
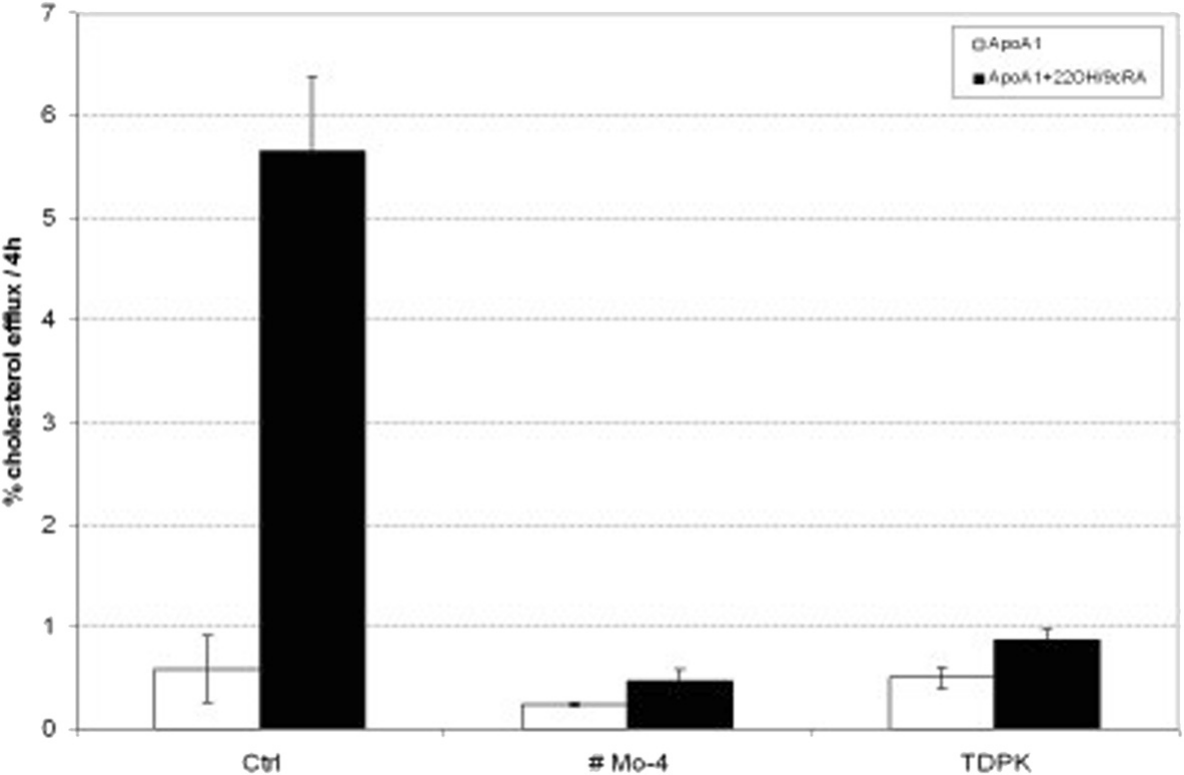
^**3**^ **H-cholesterol efflux to Apo A-I in cultured skin fibroblasts.** Cells were incubated under basal conditions (white bars) and in the presence of 22-hydroxycholesterol and 9-cis-retinoic acid (22OH/cRA) (black bars) to stimulate *ABCA1* gene expression. Ctrl: healthy control cells; #Mo-4: patient with the clinical diagnosis of Tangier Disease described in this study; TDPK: control patient with Tangier Disease reported in a previous study [4].

When PCR analysis of *ABCA1* gene was performed, all exons could be amplified and sequenced with the exception of exons 32, 33 and 34. This observation suggested that the patient might be homozygous for a deletion of these exons. To test this hypothesis the genomic region spanning from intron 30 to intron 35 was amplified. This amplification generated a ~6 kb fragment from control DNA as opposed to a ~ 2.5 kb fragment from the patient’s DNA, thus indicating that the patient was homozygous for a novel ~3.5 kb deletion (Figure 1A). By using a set of specific nucleotide primers the deletion breakpoint (i.e. the junction between intron 31 and intron 34) was characterized. The deletion was generated by a recombination between two Alu sequences (Alu-SX in intron 31 and Alu-SQ2 in intron 34 respectively) (Figure 1B). At the genomic level the mutation was designated as c.4464-486_4698 + 382 Del (Ex32_Ex34del). As expected, the RT-PCR amplification of the cDNA region spanning from exons 31 to exon 35, revealed that the genomic deletion lead to the exclusion of exons 32, 33 and 34 from the mature mRNA, with no disruption of the reading frame (Additional file 1: Figure S1). The predicted translation product of this abnormal mRNA would be a protein with an in frame deletion of 77 amino acids (from residue 1489 to residue 1566 of ABCA1 protein) (p.R1489_K1566del). As shown in Figure 2, cholesterol efflux was negligible in the patient’s fibroblasts, either under basal conditions or after stimulation of *ABCA1* expression with 9-cis-retinoic acid and 22-hydroxycholesterol. Based on these results, the diagnosis of NPC was ruled out substituted with that of TD. Miglustat treatment was then interrupted. Further examinations were conducted to assess possible TD complications: the echocolor Doppler of carotid arteries showed a 3 mm thick atherosclerotic plaque in the left common carotid trunk without hemodynamic significance, the brain angio-MRI was normal. Mild corneal opacity was present at slit lamp examination.

**Figure 3:**
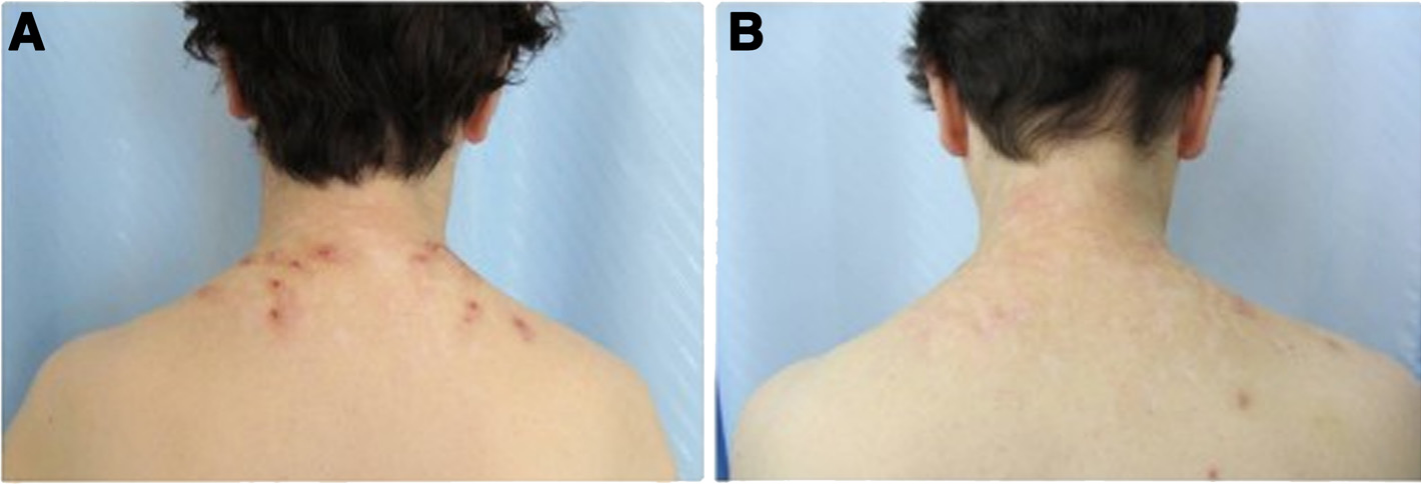
**Improvement of dorsal skin lesions after miglustat treatment.** Images showing the improvement on miglustat treatment of the dorsal skin lesions resembling prurigo nodularis of the patient with Tangier Disease described in this study. Pictures taken **A** after 7 months of miglustat discontinuation; **B** after 6 months of miglustat treatment.

**Figure 4:**
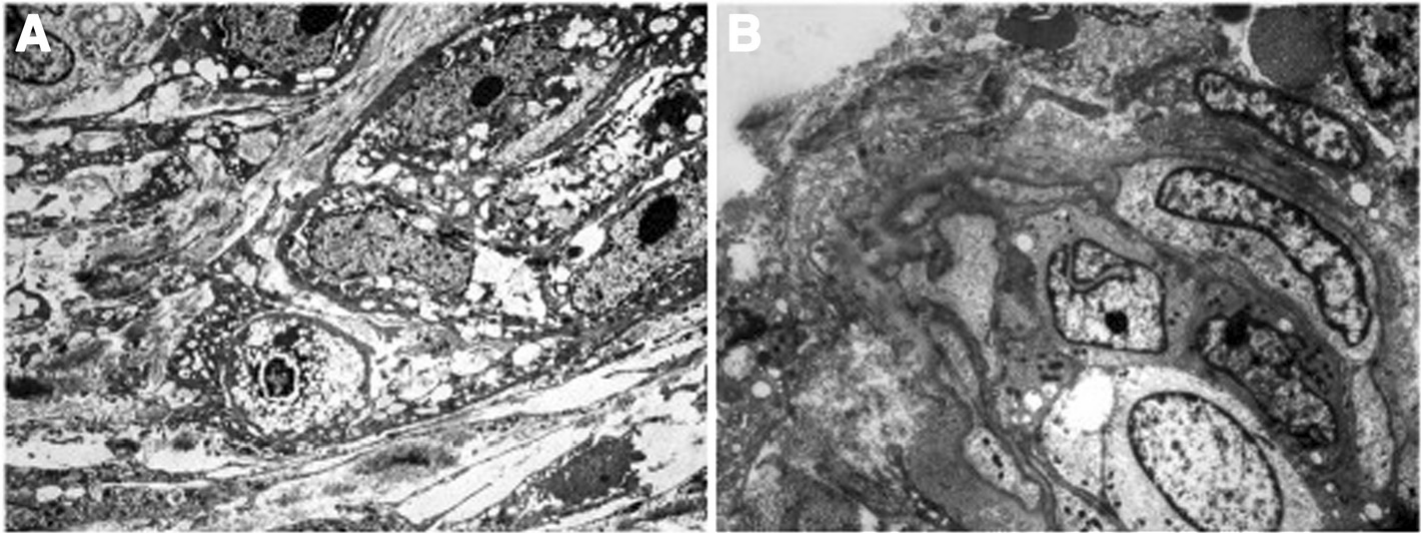
**Lipid accumulation in Schwann cells of cutaneous nerves.** Electron microphotographs showing **A** Schwann cells with accumulating cytoplasmic lipid droplets (2800 ×) and **B** well-formed redundant continuous basal lamina at the cell surface. (3500 ×).

Seven months after miglustat discontinuation skin lesions (Figure 3A) worsened, and the patient complained about reduced ability to focus attention, memory defects and slower thought processes. Skin biopsy of one dorsal nodular lesion was performed. Electron microscopy examination showed a lipid accumulation in Schwann cells of cutaneous nerves (Figure 4A and B).

These observations prompted us to hypothesize a correlation between miglustat therapy and the patient’s clinical improvement. Thus the therapy was restarted and continued for another 6 months.

During this period the patient showed a progressive amelioration of the skin lesions (Figure 3B) and a subjective improvement of general and mental wellbeing, with less fatigue and faster thought processes. No substantial improvements were noted in spleen volume, blood counts and plasma levels of HDL, ApoA1, total cholesterol and triglycerides.

## Discussion

This report describes for the first time the remission of neurocutaneous and neurological manifestations during miglustat treatment in an atypical patient with TD, initially misdiagnosed as NPC disease.

The patient had an unusual disease presentation with peripheral motor neuropathy concomitant with central nervous system symptoms, including psychomotor impairment, disabling ataxia, dysarthria and dysphagia. Interestingly, a first case of TD with predominant central nervous system findings, including dysarthria and ataxic gait (raised together with peripheral neuropathy), has been recently described in a 38-year-old female. The patient was found to be homozygous for a mutation at the 3′ acceptor splice site of intron 32 of the *ABCA1* gene [5]. Although the effect of this mutation on mRNA splicing has not been analyzed, it is likely that it causes the skipping of exon 33. If that were the case, the encoded protein would have an in frame deletion of 31 amino acids. Similarly, the abnormal ABCA1 protein of the patient described here has an in frame deletion of 77 amino acids (encoded by exons 32-34), indicating that the same domain of ABCA1 is disrupted in the two patients. Phenotypically, both patients had neurological abnormalities and did not have orange tonsils, which are generally considered a hallmark of TD. Taken together, these results indicate that the diagnosis of TD should be considered in patients showing neurological symptoms and very low HDL, even in the presence of normal tonsils.

A further unusual feature of the patient described here was the dorsal skin disease, resembling prurigo nodularis. Prurigo nodularis is an uncommon skin disease characterized by itchy nodules that can appear on any part of the body, usually beginning on the limbs. The itching and the consequent scratching of theses lesions may have a very negative impact on quality of life, and its treatment is usually challenging. The pathogenesis of these lesions remains incompletely understood. However, recent data suggest a neuropathic origin [17]. The finding of enlarged Schwann cells with lipid accumulation in the cutaneous nerves of the patient’s skin lesions suggests a direct link between prurigo nodularis and TD. It is likely that the alteration of peripheral cutaneous nerves leads to neurocutaneous manifestations. The presence of lipid accumulation in Schwann cells of patients with TD has been correlated previously with the peripheral neuropathy associated with this disease [18,19].

Neurologic manifestations of TD are considered as periodically relapsing-remitting [19]. Nevertheless, our patient, who underwent two different periods of 6-month treatment with miglustat, showed an improvement of neurological symptoms and skin lesions only when she was on treatment and relapses when the drug was discontinued.

Several studies have described a link between ABCA1 expression/function and the NPC pathway. ABCA1 is the rate-limiting step in the initial formation of plasma HDL, transferring cellular phospholipids and cholesterol to ApoAI [20]. It is well established that ABCA1 is not only localized on the cell surface, but also in late endosomes/lysosomes [21], suggesting that it plays a role in mobilizing lipids from intracellular vesicles to the extracellular ApoAI. NPC1 and NPC2, the proteins defective in NPC disease, are also located in late endosomes/lysosomes but not at the cell surface, their function being confined to intracellular lipid trafficking [22]. Furthermore, fibroblasts from TD and NPC patients share several common features: both accumulate cholesterol and sphingomyelin in late endocytic vesicles [7] and both show impaired ABCA1 dependent lipid efflux to ApoAI [6]. These findings suggest interdependence between the NPC disease pathway and ABCA1 function.

In NPC disease, the loss of NPC1 or NPC2 function leads to the accumulation of several lipids, in particular, GSL are the main species accumulating in the central nervous system [10]. Since miglustat (an inhibitor of GSL synthesis) ameliorated some of the clinical manifestations of TD in this case report, it is tempting to hypothesize that, similarly to NPC, TD patients may accumulate intracellular GSL or their derivatives, which could be implicated in the pathogenesis of the disease. Further studies to investigate this hypothesis are currently in progress.

It is also worth noting that miglustat showed a positive effect on HDL metabolism in a different lysosomal disorder, Gaucher disease. In fact, patients with Gaucher disease treated with miglustat showed significantly increased HDL and ApoAI levels [23]. The mechanism behind this effect still needs to be further investigated. However, miglustat did not affect the lipid profile in our TD patient, suggesting that it was not be able to restore the severe defect in cholesterol efflux due to the absence of a functional ABCA1 identified in this patient.

## Conclusions

Although the apparent improvement of neurological manifestations upon treatment of miglustat in a single patient cannot be taken as an indication of its effectiveness in TD, our data suggest that GSL could play a role in the pathogenesis of this disease, and that miglustat may represent a possible novel therapeutic approach for TD. The mechanisms by which miglustat ameliorates at least some clinical manifestations of Tangier disease merit further investigations.

### Consent

Written informed consent was obtained from the patient for the publication of this report and any accompanying images.

## Additional file

Additional file 1: Figure S1. Analysis of the *ABCA1* mRNA. The figure shows the RT-PCR fragment of the exon 31-exon 35 region of *ABCA1* cDNA in a control subject (lane 2) and in patient #Mo-4 (lane 3). The size difference is consistent with the deletion of exons 32, 33 and 34 in mRNA. Molecular size parkers are shown in lane 1. B) Nucleotide sequence of the exon 31-exon 35 region in *ABCA1* mRNA isolated from cultured skin fibroblasts of patient #Mo.4. The exon 31-exon 32 junction in control fibroblasts is shown in the upper panel (WT). The lower panel shows that in patient’s *ABCA*1 mRNA exon 31 is followed by exon 35 with no disruption of the reading frame.

## References

[1] Assmann G, Von Eckardstein A, Brewer HB, Scriver CR, Beaudet AL, Sly WS, Valle I (1995). Familial high density lipoprotein deficiency: Tangier disease. The metabolic and molecular bases of inherited disease.

[2] Bodzioch M, Orsó E, Klucken J, Langmann T, Böttcher A, Diederich W, Drobnik W, Barlage S, Büchler C, Porsch-Ozcürümez M, Kaminski WE, Hahmann HW, Oette K, Rothe G, Aslanidis C, Lackner KJ, Schmitz G (1999). The gene encoding ATP-binding cassette transporter 1 is mutated in Tangier disease. Nat Genet.

[3] Brunham LR, Singaraja RR, Hayden MR (2006). Variations on a gene: rare and common variants in ABCA1 and their impact on HDL cholesterol levels and atherosclerosis. Annu Rev Nutr.

[4] Fasano T, Zanoni P, Rabacchi C, Pisciotta L, Favari E, Adorni MP, Deegan PB, Park A, Hlaing T, Feher MD, Jones B, Uzak AS, Kardas F, Dardis A, Sechi A, Bembi B, Minuz P, Bertolini S, Bernini F, Calandra S (2012). Novel mutations of ABCA1 transporter in patients with Tangier disease and familial HDL deficiency. Mol Genet Metab.

[5] Negi SI, Brautbar A, Virani SS, Anand A, Polisecki E, Asztalos BF, Ballantyne CM, Schaefer EJ, Jones PH (2013). A novel mutation in the ABCA1 gene causing an atypical phenotype of Tangier disease. J Clin Lipidol.

[6] Choi HY, Karten B, Chan T, Vance JE, Greer WL, Heidenreich RA, Garver WS, Francis GA (2003). Impaired ABCA1-dependent lipid efflux and hypoalphalipoproteinemia in human Niemann-Pick type C disease. J Biol Chem.

[7] Boadu E, Francis GA (2006). The role of vesicular transport in ABCA1-dependent lipid efflux and its connection with NPC pathways. J Mol Med.

[8] Patterson MC, Vanier MT, Suzuki K, Morris JE, Carstea ED, Neufeld EB, Blanchette-Mackie EJ, Pentchev PG, Scriver CR, Beaudet AL, Sly WS, Valle D (2001). Niemann-Pick Disease type C: a lipid trafficking disorder. The metabolic and molecular bases of inherited disease.

[9] Tängemo C, Weber D, Theiss S, Mengel E, Runz H (2011). Niemann-Pick Type C disease: characterizing lipid levels in patients with variant lysosomal cholesterol storage. J Lipid Res.

[10] Zervas M, Somers KL, Thrall MA, Walkley SU (2001). Critical role for glycosphingolipids in Niemann-Pick disease type C. Curr Biol.

[11] Patterson MC, Vecchio D, Prady H, Abel L, Wraith JE (2007). Miglustat for treatment of Niemann-Pick C disease: a randomised controlled study. Lancet Neurol.

[12] Wraith JE, Vecchio D, Jacklin E, Abel L, Chadha-Boreham H, Luzy C, Giorgino R, Patterson MC (2010). Miglustat in adult and juvenile patients with Niemann-Pick disease type C: long-term data from a clinical trial. Mol Genet Metab.

[13] Patterson MC, Vecchio D, Jacklin E, Abel L, Chadha-Boreham H, Luzy C, Giorgino R, Wraith JE (2010). Long-term miglustat therapy in children with Niemann-Pick disease type C. J Child Neurol.

[14] Blanchette-Mackie EJ, Dwyer NK, Amende LM, Kruth HS, Butler JD, Sokol J, Comly ME, Vanier MT, August JT, Brady RO, Pentkev PG (1988). Type-C Niemann-Pick disease: low density lipoprotein uptake is associated with premature cholesterol accumulation in the Golgi complex and excessive cholesterol storage in lysosomes. Proc Natl Acad Sci.

[15] Fancello T, Dardis A, Rosano C, Tarugi P, Tappino B, Zampieri S, Pinotti E, Corsolini F, Fecarotta S, D’Amico A, Di Rocco M, Uziel G, Calandra S, Bembi B, Filocamo M (2009). Molecular analysis of NPC1 and NPC2 gene in 34 Niemann-Pick C Italian patients: identification and structural modeling of novel mutations. Neurogenetics.

[16] Pisciotta L, Bocchi L, Candini C, Sallo R, Zanotti I, Fasano T, Chakrapani A, Bates T, Bonardi R, Cantafora A, Ball S, Watts G, Bernini F, Calandra S, Bertolini S (2009). Severe HDL deficiency due to novel defects in the ABCA1 transporter. J Intern Med.

[17] Fostini AC, Girolomoni G, Tessari G (2013). Prurigo nodularis: an update on etiopathogenesis and therapy. J Dermatolog Treat.

[18] Pollock M, Nukada H, Frith RW, Simcock JP, Allpress S (1983). Peripheral neuropathy in Tangier disease. Brain.

[19] Cai Z, Blumbergs PC, Cash K, Rice PJ, Manavis J, Swift J, Ghabriel MN, Thompson PD (2006). Paranodal pathology in Tangier disease with remitting-relapsing multifocal neuropathy. J Clin Neurosci.

[20] Oram JF (2002). ATP-binding cassette transporter A1 and cholesterol trafficking. Curr Opin Lipidol.

[21] Chen W, Wang N, Tall AR (2005). A PEST deletion mutant of ABCA1 shows impaired internalization and defective cholesterol efflux from late endosomes. J Biol Chem.

[22] Higgins ME, Davies JP, Chen FW, Ioannou YA (1999). Niemann-Pick C1 is a late endosome-resident protein that transiently associates with lysosomes and the trans-Golgi network. Mol Genet Metab.

[23] Puzo J, Alfonso P, Irun P, Gervas J, Pocovi M, Giraldo P (2010). Changes in the atherogenic profile of patients with type 1 Gaucher disease after miglustat therapy. Atherosclerosis.

