# Supplementary figures and images for: Effects of miglustat treatment in a patient affected by an atypical form of Tangier disease
Source: Orphanet J Rare Dis. 2014 Sep 18;9:143. doi: 10.1186/s13023-014-0143-3 (PMC4172812; doi:10.1186/s13023-014-0143-3)

**
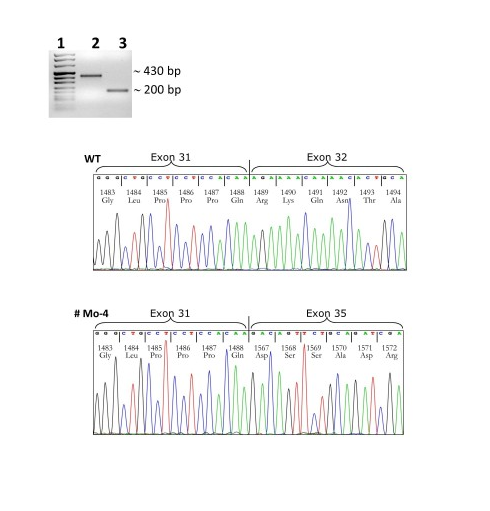
**

Supplement: Additional file 1: Figure S1. — Analysis of the ABCA1 mRNA. The figure shows the RT-PCR fragment of the exon 31-exon 35 region of ABCA1 cDNA in a control subject (lane 2) and in patient #Mo-4 (lane 3). The size difference is consistent with the deletion of exons 32, 33 and 34 in mRNA. Molecular size parkers are shown in lane 1. B) Nucleotide sequence of the exon 31-exon 35 region in ABCA1 mRNA isolated from cultured skin fibroblasts of patient #Mo.4. The exon 31-exon 32 junction in control fibroblasts is shown in the upper panel (WT). The lower panel shows that in patient’s ABCA1 mRNA exon 31 is followed by exon 35 with no disruption of the reading frame. [file 13023_2014_143_MOESM1_ESM.doc]
